# Applying precision medicine to the diagnosis and management of endocrine disorders

**DOI:** 10.1530/EC-22-0177

**Published:** 2022-08-05

**Authors:** Martin Bidlingmaier, Helena Gleeson, Ana-Claudia Latronico, Martin O Savage

**Affiliations:** 1 Medizinische Klinik und Poliklinik IV, LMU Klinikum, Ludwig-Maximilians University, Munich, Germany; 2 Department of Endocrinology, Queen Elizabeth Hospital, Birmingham, UK; 3 Department of Internal Medicine, Discipline of Endocrinology and Metabolism, Sao Paulo Medical School, University of Sao Paulo, Sao Paulo, Brazil; 4 Centre for Endocrinology, William Harvey Research Institute, Barts and the London School of Medicine and Dentistry, London, UK

**Keywords:** adolescent, biomarkers, child, early diagnosis, endocrine diagnosis, genetic testing, growth hormone, precocious puberty, precision medicine, transitional care

## Abstract

Precision medicine employs digital tools and knowledge of a patient’s genetic makeup, environment and lifestyle to improve diagnostic accuracy and to develop individualised treatment and prevention strategies. Precision medicine has improved management in a number of disease areas, most notably in oncology, and it has the potential to positively impact others, including endocrine disorders. The accuracy of diagnosis in young patients with growth disorders can be improved by using biomarkers. Insulin-like growth factor I (IGF-I) is the most widely accepted biomarker of growth hormone secretion, but its predictive value for recombinant human growth hormone treatment response is modest and various factors can affect the accuracy of IGF-I measurements. These factors need to be taken into account when considering IGF-I as a component of precision medicine in the management of growth hormone deficiency. The use of genetic analyses can assist with diagnosis by confirming the aetiology, facilitate treatment decisions, guide counselling and allow prompt intervention in children with pubertal disorders, such as central precocious puberty and testotoxicosis. Precision medicine has also proven useful during the transition of young people with endocrine disorders from paediatric to adult services when patients are at heightened risk of dropping out from medical care. An understanding of the likelihood of ongoing GH deficiency, using tools such as MRI, detailed patient history and IGF-I levels, can assist in determining the need for continued recombinant human growth hormone treatment during the process of transitional care.

## Introduction

The term ‘precision medicine’ can be defined as a pathway that employs technologies (digital tools) and knowledge of a patient’s individual characteristics (including their genetic makeup) to guide individually tailored diagnostic methods and treatments (1, 2). The aim is to guide healthcare decisions to ensure the delivery of the most effective treatment for a patient, with a focus on data, analytics and information (3), and to use data generated during clinical care to inform further research in this area (4). Precision medicine is reasonably well advanced in some therapeutic areas, particularly oncology, but its use in the day-to-day care of patients with endocrine disorders is currently more limited. Nevertheless, precision medicine has the potential to markedly improve this area of healthcare.

This review article is based on a webinar that was broadcast on 21 October 2021, in which the four authors presented information on the use of precision medicine in the diagnosis and management of young patients with endocrine disorders to participating clinicians and answered questions related to their presentations. The aims of the webinar, and thus this review, were three-fold: (i) to explain how precision medicine can contribute to accurate biochemical diagnosis of growth disorders; (ii) to discuss how to utilise genetic analyses appropriately to confirm pathogenesis and enable personalised clinical management; and (iii) to review approaches to the integration of precision medicine practices and tools into transitional care for patients with growth hormone (GH) deficiency. Data from the published literature on linear growth in children with GH deficiency, disorders of puberty, genetic analysis, paediatric adrenal tumours and transitional care of the GH-deficient patient are reviewed, and the authors provided comments and recommendations based on their extensive clinical experience in this area.

## Role of precision medicine in accurate biochemical diagnosis of growth disorders

GH deficiency can be used to illustrate the importance of accurate biochemical diagnosis in the management of endocrine disorders. A biomarker (or biological marker) has been defined by the Biomarkers Definitions Working Group as ‘*a characteristic that can be objectively measured and evaluated as an indicator of normal biological processes, pathogenic processes, or the pharmacological response to a therapeutic intervention*’ (5). Biomarkers can act as: (i) clinical endpoints, reflecting how the patient feels, functions or survives; or (ii) surrogate endpoints, which are intended to substitute for a clinical endpoint that may take longer than the study duration to become apparent and which are suitable for predicting the expected benefit (or lack of benefit) based on scientific evidence (5).

### IGF-I as a biomarker for GH status

Many components in the blood are sensitive to GH and so could be candidate biomarkers for GH levels and, thus, be used to determine GH deficiency and the need for treatment. In the 1990s, insulin-like growth factor (IGF)-binding protein 3 (IGFBP-3) was often used as an indicator of GH levels, particularly in children younger than 8 years, probably because of the difficulties at that time of measuring IGF-I at low concentrations. However, IGFBP-3 is a less sensitive indicator of change in GH status than is IGF-I. More recently, IGF-I has become the standard biomarker for GH. Indeed, in 2017, a workshop of the Growth Hormone Research Society discussed clinical endpoints, surrogate endpoints and biochemical biomarkers during GH treatment of adults and children with GH deficiency (6) and concluded that IGF-I is still the most widely accepted biochemical biomarker for GH action. The workshop also concluded that IGF-I can contribute to most of the clinical and surrogate endpoints that are used to diagnose, treat and monitor patients.

### Considerations when using IGF-I as a biomarker

While a correlation between GH and IGF-I exists, this correlation is only of modest strength in both adults and children (7, 8), indicating large biological variability in the relationship between GH secretion and IGF-I levels. This was demonstrated in a study in which healthy individuals (*n*  = 20) were administered bolus injections of recombinant human GH (rhGH) (9). As expected, the IGF-I level increased in all study participants after administration of rhGH but this occurred regardless of the dose and route of administration and with marked variability between the individuals.

The results of studies that assessed the use of IGF-I to predict response to rhGH treatment suggest only minimal predictive value (10, 11, 12). The high biological variability of IGF-I is a result of the numerous factors, in addition to GH secretion, that modify the concentration of IGF-I, such as malnutrition, liver dysfunction, diabetes mellitus, hypothyroidism, and sex steroid levels (13).

As a further complication, laboratory practices and assays may contribute to the variability in hormone measurements. An external quality assessment scheme (RfB Germany) (14) conducted to investigate the comparability of laboratory GH measurements found marked variability among several hundred laboratories that were provided with the same two samples to analyse GH, with levels from 3 to 12 ng/mL reported. The assay methods used also significantly affected the GH concentration determined. Wagner *et al*. (15) analysed GH in samples from stimulation tests in children and, as a reference, used the Immunodiagnostic Systems iSYS method to calculate a threshold that discriminated GH-deficient from GH-sufficient children in their cohort, finding a cut-off point of 7.09 ng/mL. They then analysed the same samples using other methods and found that the cut-off point varied from just over 4 ng/mL to almost 8 ng/mL, depending on the assay (see Table 1). Similar variability between laboratories was seen in the RfB scheme when assessing IGF-I levels, with values of between 200 ng/mL and 1000 ng/mL reported for one and the same sample, and a very significant impact of the analytical method used.

**Table 1:** Assay variation in cut-off limits to differentiate GH-deficient from GH-sufficient children in GH stimulation tests, using the iSYS test as the reference (15). Reproduced, with permission, from Wagner *et al.* 2014, *European Journal of Endocrinology*, vol 171 page 393. Copyright 2014 European Society of Endocrinology (15).

Assay	GH cut-off limit (ng/mL)
Immulite 2000 (Siemens)	7.77
AutoDELFIA (Perkin-Elmer)	7.44
*iSYS (Immunodiagnostic Systems) - reference*	*7.09*
Liason (DiaSorin)	6.25
RIA (in-house Tuebingen)	5.28
Dxi (Beckmann-Coulter)	5.15
ELISA (Mediagnost)	5.14
BC-IRMA (Beckmann-Coulter)	4.32

GH, growth hormone.

**Figure 1:**
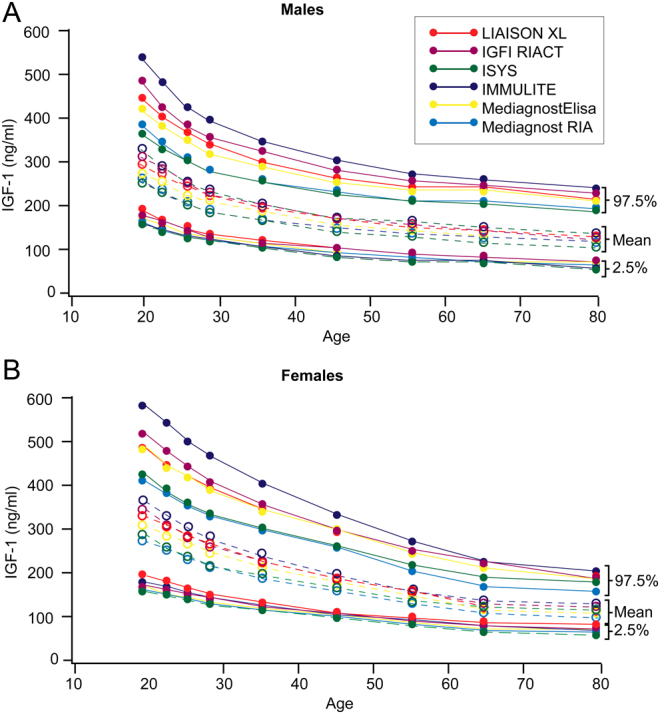
Reference intervals for (A) males and (B) females according to the age intervals of six insulin-like growth factor I (IGF-I) immunoassays. Lower limits (2.5th percentile) and upper limits (97.5th percentile) of the normal range are drawn as full lines and means as dotted lines. Data from Chanson *et al.* (17).

One reason for the variation between IGF-I assays is the use of different methods to avoid interference from IGFBPs. More specifically, assays differ in the concentrations of IGF-II added (due to its expense) and in the extraction methods in the IGF-II displacement assay, which is the gold standard for measuring IGF-I levels. Because of this variability, it is mandatory to have reference intervals for interpretation that are specific for each assay method. This was demonstrated by Ranke *et al*. (16) who compared four different commercially available IGF-I immunoassays, using the same 700 samples from healthy adults in each assay. They calculated reference intervals based on the results of one assay and found that these intervals could not be translated to the other three assays. Similarly, Chanson *et al.* (17) found significant variation in the upper reference intervals across six different commercial assay kits that they used to measure IGF-I levels in healthy adults (*n*  = 911), keeping the statistical method constant (see Fig. 1). This highlights the importance of calculating reference intervals for the specific assay that is being used, rather than using intervals reported in the literature. It has been suggested that conversion of IGF-I levels into sds could be used to solve the problem of reference interval variation across assays. However, large variability in the reference ranges of commercially available IGF-I assays, particularly in the upper limits of the reference range, has been found, with only moderate average concordance found across eight immunoassays (18). These variations in assay agreement were independent of the use of mass units or SDS. Given this variability, if possible, patients with GH disorders should be monitored using the same IGF-I assay at each assessment point (18).

Differences in reference intervals may also be the result of factors such as sample size, statistical methodology (e.g. 2.5th percentile vs –3 s.d.) and type of age calculation (pubertal development stage (Tanner stage) vs chronological age) (19). Further, it is important to consider that, even in healthy populations, IGF-I levels of some individuals will naturally fall outside the reference ranges of each assay, which is defined as the central 95% interval. There is also a need to consider how IGF-I levels are reported, with both ng/mL and sds having a place, although the usefulness and accuracy of the latter depend on the quality of the underlying data set used to define the reference intervals.

Finally, when using IGF-I levels as a biomarker during treatment, it is important that the timing of blood sampling in relation to treatment administration is consistent; this will become increasingly important with the potential introduction of long-acting rhGH formulations. For example, if the sample is taken shortly after the injection of a long-acting rhGH, the IGF-I levels will be high (peak levels), while if it is taken 6 days after the injection or shortly before the next injection, the IGF-I levels will be much lower (trough levels). Clinicians, therefore, need to familiarise themselves with the pharmacokinetic and pharmacodynamic characteristics of long-acting rhGH formulations and to interpret IGF-I levels with care in this situation.

The above points illustrate that while IGF-I remains the most widely accepted biomarker of GH action (apart from GH itself), its predictive value for response to rhGH treatment is modest. Further, the IGF-I assay method, the need for assay-specific reference intervals and the impact of biological confounders need to be accounted for when considering IGF-I as a component of precision medicine in the management of GH deficiencies. In addition, IGF-I levels need to be considered in the context of the observed clinical efficacy of rhGH treatment in order to ensure the most benefit for patients.

## Value of genetic analyses in precision medicine for endocrine disorders

Genetic discoveries have contributed to our understanding of the mechanisms responsible for many diseases, both rare and common. This, in turn, has allowed the development of novel strategies, including therapeutics, dietary/behaviour modifications, prophylactic interventions and surveillance programmes, that are tailored to individual patients or subgroups of patients with the same genetic predisposition and disease characteristics, which aim to improve patient outcomes. Examples include the development of new medications based on targeted treatment for cystic fibrosis and spinal muscular atrophy, a phenylalanine-free diet to manage phenylketonuria, an implantable cardioverter-defibrillator to correct cardiac arrhythmias associated with Brugada syndrome and early initiation of colonoscopic surveillance to detect bowel cancer in patients with Lynch syndrome (20). Genetic approaches to diagnosis and treatment have also been investigated in the context of several paediatric endocrine disorders.

### Central precocious puberty

#### Monogenic CPP

Using next-generation sequencing analysis, the first monogenic causes of central precocious puberty (CPP) were identified in patients previously classified as having an idiopathic disease form. Four distinct causes have been recognised (see Table 2) (21). Each cause differs in frequency, clinical features and outcomes.

**Table 2:** Known monogenic causes of CPP.

Gene product	Mutation	Frequency	Clinical features	Outcome
Kisspeptin receptor (*KISS1R*)	Gain of function	Very rare (isolated case)	Undefined	Unknown
Kisspeptin (*KISS1*)	Gain of function	Very rare (isolated case)	Very early puberty (first year of life)	Unknown
Makorin RING finger protein 3 (*MKRN*3)	Loss of function	Frequency in hereditary CPP (33–46%) with paternal transmission	Typical CPP, with adequate response to GnHR analogue treatment	Severe mutations associated with higher basal LH and advanced bone age
Delta-like 1 homologue (*DLK1*)	Loss of function	Very rare in hereditary CPP	CPP, very early menarche, PCOS	Metabolic abnormalities in adulthood

CPP, central precocious puberty; GnHR, gonadotropin hormone-releasing hormone; LH, luteinising hormone; PCOS, polycystic ovary syndrome.

#### MKRN3 mutations

Loss-of-function mutations of the Makorin RING finger protein 3 gene (*MKRN3*) are the most frequent genetic defects associated with CPP, appearing in up to 46% of affected European families (22). Clinically, *MKRN3* mutations result in features similar to those of typical CPP and are associated with a satisfactory response to gonadotropin hormone-releasing hormone (GnRH) analogue treatment. However, this type of mutation can affect the phenotype. An analysis of 716 multi-ethnic patients with familial or idiopathic CPP found that 71 patients (45 girls and 26 boys from 36 families) had 18 different loss-of-function *MKRN3* mutations (23). Eight of these mutations were classified as severe, including nonsense and frameshift mutations, and were identified in approximately 70% of the patients with *MKRN3* mutations. The patients with severe *MKRN3* mutations had a greater bone age advancement compared with patients with missense mutations and had higher basal luteinising hormone (LH) levels at the time of presentation.

**Figure 2:**
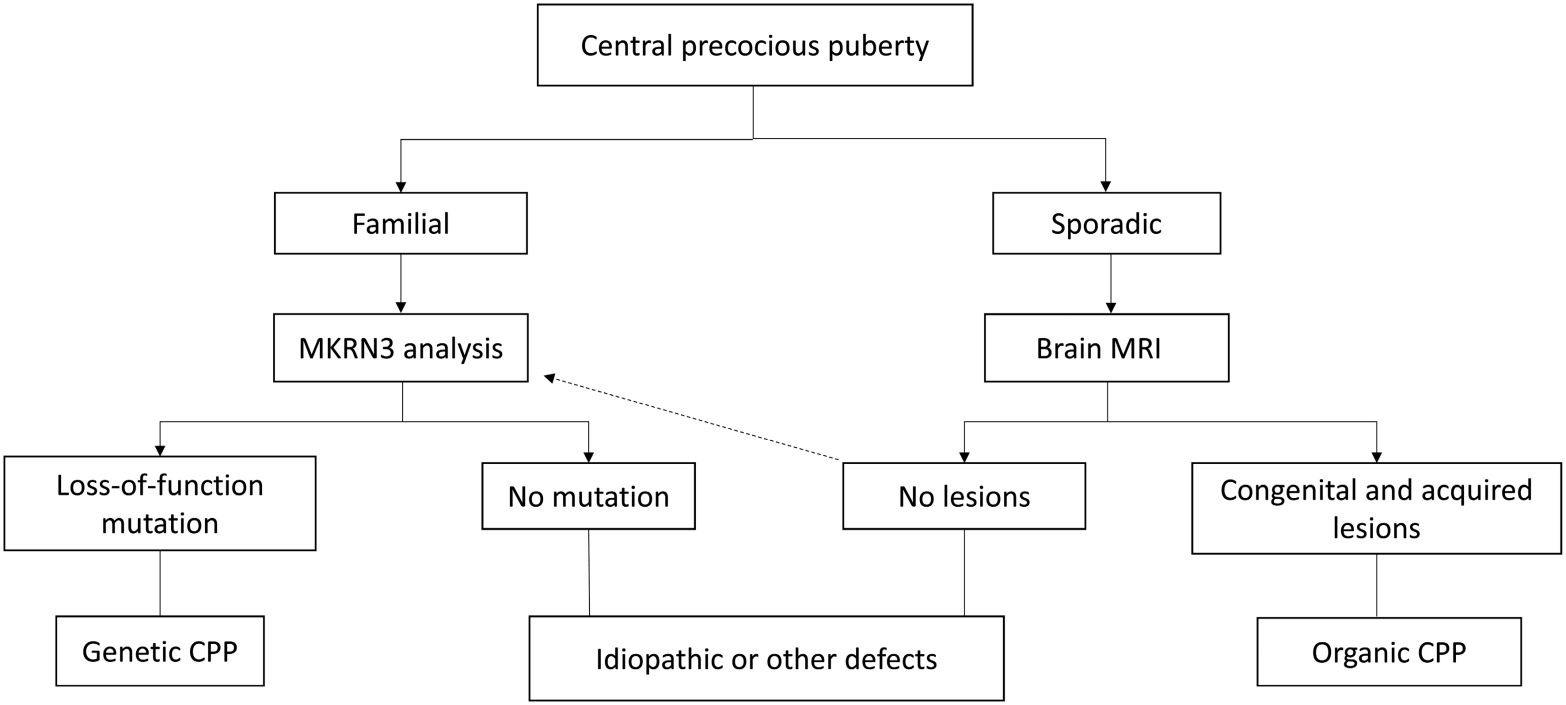
Indications for genetic testing in the diagnosis of central precocious puberty (CPP). Data from Latronico *et al.* (24). MKRN3, Makorin RING finger protein 3. The dotted arrow indicates the possibility of performing a genetic test (*MKRN3* gene analysis) after a normal MRI in children with sporadic CPP.

As a result of the high frequency of *MKRN3* mutations in familial cases of CPP, genetic testing is being increasingly used in the clinical investigation of the disorder (see Fig. 2). In familial cases, genetic tests could precede brain MRI, with such imaging potentially being delayed or even avoided completely in patients who carry *MKRN3* mutations (24). Despite the recent advances in understanding of the genetic basis of CPP, its treatment is still based on GnRH analogues. These agents have shown similar efficacy and long-term outcomes in children with CPP due to *MKRN3* mutations and in those with idiopathic CPP (25).

#### DLK1 mutations

The first loss-of-function mutations of the delta-like 1 homologue gene (*DLK1*) in families with CPP were described recently (26, 27). Metabolic abnormalities such as overweight, hyperlipidaemia and type 2 diabetes mellitus were more prevalent in the patients with familial CPP who had the *DLK1* mutations (*n*  = 10), compared with the idiopathic CPP group (*n*  = 20). Such patients have a different prognosis from those patients without the *DLK1* mutation due to an elevated cardiovascular risk profile, suggesting a precise, tailored approach to management in these patients that focuses on this risk profile.

#### Syndromic CPP

A cohort of 197 patients with CPP and no structural brain lesion or monogenic causes of CPP underwent a detailed clinical evaluation, which identified 36 patients (18%) who had a syndromic CPP that was associated with multiple abnormalities (28). This subgroup underwent methylation analysis of candidate regions, chromosomal microarray analysis and, in a small subset, whole-exome sequencing. Rare genetic abnormalities, including Temple syndrome, William–Beurens syndrome and SHOX syndrome, were evident in 12 patients (33%) from the selected subgroup with syndromic signs. In these patients with syndromic CPP, metabolic, growth and neurocognitive phenotypes were the most prevalent, again providing insight into the appropriate management for this subgroup. A precise genetic diagnosis of syndromic CPP does impact treatment and prognosis, favouring the combination of GnRH analogues and GH replacement in some diseases, such as Temple syndrome and SHOX syndrome, both conditions characterised by post-natal growth failure. The distinct causes of syndromic CPP without anatomical lesions in the CNS are described in Table 3 (29).

**Table 3:** Syndromic central precocious puberty without CNS lesions.

Syndrome	Critical region	Main molecular diagnosis	Prevalence of CPP (%)	Other main clinical features	Putative mechanism(s) or gene involved in CPP
Temple syndrome	14q32.2	UPD(14)mat or *DLK1/MEG3*:IG-DMR hypomethylation or 14q32.2 paternal deletion	80–90	Prenatal and postnatal growth failure, hypotonia, small hands and/or feet, obesity, motor delay	*DLK1*
Prader–Willi syndrome	15q11-q13	15q11-q13 paternal deletion or UPD(15)mat	4	Hypotonia, obesity, growth failure, cognitive disabilities, hypogonadism	*MKRN3*
Silver–Russell syndrome	11p15.5	*IGF2/H19*:IG-DMR hypomethylation	Overall: 5-15 UPD(7)mat: likely higher prevalence	Prenatal and postnatal growth retardation, relative macrocephaly, prominent forehead, body asymmetry, feeding difficulties	11p15.5 defects: not established
	Chromosome 7	UPD(7)mat			UPD(7)mat: possible imprinted or recessive factors to be elucidated
Williams–Beuren syndrome	7q11.23	Hemizygous 7q11.23 deletion	3-18	Distinct facial features, cardiovascular disease, short stature, intellectual disability, hypersociability	Contiguous gene syndrome; CPP mechanism remains unclear
Xp22.33 deletion (*SHOX* region)	Xp22.33	Xp22.33 deletion with pseudo-autosomal dominant inheritance, involving *SHOX*	Rare cases	*SHOX* phenotypes: body disproportion, short stature, Madelung deformity	CPP mechanism remains unclear
Xp11.23-p.11.22 duplication syndrome	Xp11.23-p11.22	Xp11.23-p11.22 duplication with X-linked dominant inheritance	70 (girls) 11 (boys)	Intellectual disability, speech delay, EEG abnormalities, excessive weight, skeletal anomalies	Contiguous gene syndrome; CPP mechanism remains unclear
Rett syndrome	Xq28	*MECP2* loss-of-function mutations or Xq28 duplication involving *MECP2*	Rare cases	Neurodevelopmental phenotypes, intellectual disability, autism	*MECP2*
X-linked intellectual developmental disorder Snijders Blok type	Xp11.4	X-linked dominant *de novo* mutations in *DDX3X* affecting females	13 (girls)	Intellectual disability, developmental delay, hypotonia, behavioural problems, movement disorders, skin abnormalities	*DDX3X* (29)
Kabuki syndrome	12q13.12	Loss-of-function mutations in *KMT2D*	Premature thelarche: 40 CPP: uncommon	Neurodevelopmental phenotypes, typical distinct face, short stature, multiple anomalies	Possible downregulation of oestrogenic receptor activation
Mucopolysaccharidosis type IIIA or Sanfilippo disease	17q25.3	Homozygous or compound heterozygous mutations in *SGSH*	Very rare	Severe neurological deterioration, visceromegaly, skeletal abnormalities	Possible accumulation of glycosaminoglycans triggering GnRH

CPP, central precocious puberty; DDX3X, DEAD-box helicase 3; DLK1, delta-like 1 homologue; GnRH, gonadotropin hormone-releasing hormone; IG-DMR, intergenic differentially methylated regions; IGF2, insulin-like growth factor 2; KMT2D, lysine methyltransferase 2D; MECP2, methyl-CpG binding protein 2; MEG3, maternally expressed gene 3; MKRN3, Makorin RING finger protein 3; SGSH, N-sulfoglucosamine sulfohydrolase; UPD(7)mat, maternal uniparental disomy of chromosome 7; UDP(14)mat, maternal uniparental disomy of chromosome 14; UDP(15)mat, maternal uniparental disomy of chromosome 15.

### Adrenocortical tumours and *p53* mutations

Children from the southern regions of Brazil have a higher incidence of adrenocortical tumours than children who live in other parts of the world, initially thought to be due to pesticides and other industrial chemicals. However, in 2001, it was found that Brazilian children with adrenocortical tumours have a high frequency of inherited missense mutations of the *p53* gene (30). Carriers of the founder *R337H* mutant allele have an increased risk of developing adrenocortical tumours and other types of malignancies, including breast carcinoma, soft-tissue sarcoma, osteosarcoma, choroid plexus carcinoma, and thyroid and lung cancers (31). More recently, whole-genome sequencing identified a variant in another gene – a tumour suppressor, *XAF1* – in a subset of patients who carried the *R337H* mutation (32). The compound mutant haplotype was enriched in patients with cancer, conferring risk for sarcoma and other tumours. Functional analysis demonstrated that the WT *XAF1* enhances transactivation of the WT in the mutant *p53* variant, whereas the *XAF1* mutation is markedly attenuated in this regard. The co-segregation of two mutations leads to a more aggressive cancer phenotype than a *p53* mutation alone. Knowledge of the implications of hypomorphic *p53* mutations allows for intervention in the form of genetic counselling and clinical management. Radical oncologic surgery (in block) followed by abdominal radiotherapy and chemotherapy (mitotane) can be applied more frequently and earlier in the treatment course in those cases with an aggressive cancer genotype. In addition, the possibility of the development of a second cancer (especially sarcoma) must be rigorously evaluated by short intervals between medical consultations and imaging studies in this group of paediatric patients with an unfavourable genotype, as well as in their affected close relatives.

### Isolated FSH deficiency

In another interesting case, the clinical, hormonal and molecular features of a female adolescent with selective follicle-stimulating hormone (FSH) deficiency were reported (33). The patient was a 16-year-old girl with primary amenorrhoea and poor breast development due to isolated FSH deficiency. She was found to have a homozygous nonsense mutation in amino acid position 76 of the FSH β-subunit gene. The induction of fertility using recombinant human FSH (rhFSH) in a woman with the same condition of isolated FSH deficiency was later reported by Kottler *et al.* (34). In response to a 15-day treatment with FSH alone, sonography revealed multi-follicular development in the ovaries, and circulating levels of oestradiol and inhibin B were dramatically increased. Serum LH initially decreased and then increased, inducing multi-ovulation associated with supra-physiological progesterone levels. This case illustrates the use of genetic analysis to assist in the identification of appropriate treatment, in this case, rhFSH for ovulation induction in a patient with a rare form of FSH deficiency.

### Preclinical diagnosis of pubertal disorders

Genetic discoveries also hold potential for allowing very early diagnosis in children with pubertal disorders.

In 2016, we reported the case of an asymptomatic girl, who was carrying an *MKRN3* mutation that was detected in childhood and was followed until the development of pubertal signs (35). The patient was screened at the age of 4 years because her sister had developed CPP at 6 years of age and was found to harbour an *MKRN3* frameshift mutation at codon 161, inherited from their asymptomatic father. During follow-up, the asymptomatic girl initially developed increased growth velocity at 6 years (9 cm/year), followed by slightly increased basal LH level and clinical thelarche with rapid progression between 6.3 and 6.7 years. The finding of a loss-of-function mutation plus a positive family history allowed the early diagnosis of CPP and supported the initiation of treatment with an GnRH analogue.

#### Testotoxicosis

Similarly, preclinical diagnosis of testotoxicosis is now possible using genetic tests. Testotoxicosis is a rare, autosomal-dominant, peripheral precocious puberty. It is usually recognised by progressive virilisation, linear growth acceleration, skeletal maturation and pubertal testosterone levels. We have described the clinical and hormonal follow-up of a boy with testotoxicosis, who was initially diagnosed by molecular analysis (36). At age 10 months, the patient was referred because his older brother had a diagnosis of familial testotoxicosis found to be due to a missense mutation of the LH-chorionic gonadotropin receptor (*LHCGR*) gene. The same mutation was identified in the asymptomatic boy, who presented with an increase in testosterone level but without any clinical features. As a result, he was treated with an anti-androgen therapy at the age of 1.1 years, which resulted in him growing to normal adult height.

Therefore, genetic analysis (supported by large international genetic banks to differentiate pathogenic from benign gene variants) has much to offer. It is able to assist in early diagnosis, facilitate treatment decisions, guide genetic counselling (including screening of family members) and allow prompt intervention in children with pubertal disorders.

## Integration of precision medicine into transitional care of patients with growth hormone deficiency

Replacement therapy with rhGH, in addition to promoting linear growth in childhood and adolescence, has important effects in GH-deficient adults, such as optimisation/maintenance of normal body composition (fat and lean body mass), bone health and metabolism. Continuation of rhGH therapy into adulthood may, thus, be indicated in some adolescents who have received this treatment. Unfortunately, this period of transition is often when adolescents drop out of follow-up medical care, highlighting the importance of clinical programmes to allow the effective transfer of rhGH-treated adolescents from paediatric to adult endocrine services (37).

### Transition and transfer

Transition is a process that spans from adolescence into young adulthood and refers to a ‘*multifaceted active process, that attends to the medical, psychosocial, and educational vocational needs of adolescents as they move from child-focused to the adult-focused healthcare system*’ (38). ‘Transfer’ is an event that happens within transition. Given the multidimensional nature of transition, precision medicine holds great potential for improving the process.

#### Planning for transition

Effective planning for transition is the first step in ensuring the orderly transfer of care, and it should begin at around age 13–14 years. In the United Kingdom, the ‘Ready Steady Go’ system is often used for this purpose (39). The system was first developed for use in rheumatology patients (40) and is a step-wise process that assists with gradually transitioning the patient from adolescent to adult services. The first step (‘Ready’) should be delivered in early adolescence, followed by subsequent steps (‘Steady’ and ‘Go’) that the young person progresses through at their own pace. The aim is to ensure that the young person’s needs are being met to enable them to successfully transition/transfer to adult services. It is vital that the structure of the adult service allows young patients to meet the adult team prior to transfer, to develop trusting relationships and have safeguards in place to ensure that they have a point of access in the adult service.

The healthcare professionals involved in providing information to the patient must complement any digital technology that they use and complex data generated from it (such as genetic analysis) with one-on-one interactions with the patient. Further, the presentation of information must be at a level that the patient can understand, which may include the involvement of a carer who acts as the young person’s advocate, particularly for patients who have learning difficulties.

#### Transition Readiness Assessment Questionnaire and smartphone apps

There are a number of tools available to assess a young person’s readiness to transition, including the Transition Readiness Assessment Questionnaire (TRAQ). This has recently been used in endocrinology to compare young people with Turner syndrome to those with type 1 diabetes and revealed that those with Turner syndrome have less autonomy in their healthcare (41). In addition, mobile technologies, such as smartphone apps (e.g. Tiny Medical Apps’ Digital Health Passport app), have been developed that can assist young people in self-managing their condition. These provide information about the patient’s illness and the investigations they are likely to undergo and advise them on how to manage their health care and when to access routine care or emergency care, thereby increasing the likelihood that they will access health services when they need to.

### Reassessment of GH status

The accurate identification of patients with likely ongoing GH deficiency who will require continued rhGH replacement is important and involves the reassessment of GH status. Essentially, we recommend following published guidelines (42). The use of imaging, such as MRI of the pituitary, can guide this process. Patients with an ectopic posterior pituitary (which is a very clear abnormality on MRI) are more likely to have severe GH deficiency, while those with a normally placed posterior pituitary on MRI are more likely to have a normal GH status (43). Studies have shown that, depending on the test and cut-off used, between 66% and 85% of patients with no structural pituitary abnormality on MRI will have adequate GH secretion when tested in late adolescence or adulthood (44).

Criteria have also been defined that identify patients who do not require reassessment of GH status because of a high likelihood of ongoing severe GH deficiency, that is, those with: (i) multiple pituitary hormone deficiencies (i.e. three or more) plus low serum IGF-I levels (≤2 SDS); (ii) genetic defects affecting the hypothalamic–pituitary axis; or (iii) lesions of the hypothalamic–pituitary area (42). These patients can continue GH therapy without interruption during transition.

In 2005, the European Society for Paediatric Endocrinology published a consensus statement to guide the evaluation of rhGH-treated young people at the end of linear growth. It was recommended that patients be categorised into two groups – with a low or a high likelihood of severe GH deficiency (37). The former group should be more robustly investigated for ongoing GH deficiency, with both low GH levels on a stimulation test and low IGF-I levels needed to demonstrate eligibility for ongoing rhGH treatment into late adolescence and young adulthood. If both these tests are normal, the patient should not receive further rhGH treatment and may not require ongoing follow-up, unless there is a risk of evolving endocrinopathy. In patients with a high likelihood of severe GH deficiency, a single assessment can be used to determine GH status, for example, IGF-I level alone or GH stimulation test alone. Patients who are likely to be at high risk of ongoing GH deficiency are those with hypothalamic–pituitary area lesions, who have received high-dose (>35 Gy) cranial radiotherapy, or who have congenital GH deficiency (although dependent on MRI appearance). Patients with idiopathic GH deficiency and a normal pituitary are at low risk.

More recent guidelines, from the American Association of Clinical Endocrinologists and American College of Endocrinology (published in 2019) (42), recommend that no reassessment of GH status is required for adolescents with idiopathic GH deficiency and an IGF-I level of ≥0 SDS. As there is no indication that these patients will progress and develop other pituitary hormone deficiencies, they can be discharged, with no transfer to adult services required. In patients with organic isolated GH deficiency, the number of GH tests should be guided by clinical suspicion of the high or low risk of ongoing GH deficiency.

#### Biochemical cut-off for defining persistent GH deficiency

Another area in which precision medicine has a role is the determination of the cut-off for defining ongoing and persistent GH deficiency. In childhood, the cut-off is classically a GH level of <10 ng/mL, although this has been lowered to <7 ng/mL in some centres. Severe GH deficiency in adults is defined as a GH level of <3 ng/mL. In late adolescence, when GH levels peak, the choice of a mid-point is intuitive. Indeed, a study of adolescents aged 17–19 years (*n*  = 79) showed that a value of approximately 6 ng/mL (in response to an insulin tolerance test (ITT)) identified a group at high risk of GH deficiency (45). However, pragmatically, consensus statements have favoured using the adult criteria (ITT-induced level of <3 ng/mL) in adolescents (42). The use of BMI to allow more precision in diagnosis has also been suggested, with GH deficiency defined by a GH stimulation test GH level of 3 ng/mL in those of normal weight (BMI <25 kg/m^2^) or those who are overweight (BMI 25–30 kg/m^2^) with a high pre-test probability of GH deficiency but defined by a level of 1 ng/mL in patients who are overweight with a low pre-test probability and those who are obese (BMI >30 kg/m^2^) (42). Future real-world research assessments should be based on precision factors such as MRI appearance, baseline IGF-I and pre-existing multiple pituitary hormone deficiencies to gauge the strength of these factors in diagnostic accuracy and responses to rhGH therapy.

Precision is also needed when considering the risks and benefits of rhGH therapy. This can be illustrated by the effects on bone health. Högler *et al*. (46) reviewed the evidence relating to the impact of GH deficiency on bone and, in particular, reviewed all the available fracture studies. The fracture prevalence and incidence studies demonstrated evidence of increased fracture risk in those with adult-onset multiple pituitary hormone deficiency, but there was insufficient evidence of increased risk in those with isolated GH deficiency of either childhood or adult onset. In another group of studies assessing untreated, longstanding, often genetic severe GH deficiency, there was no increased risk of fractures in those with isolated childhood-onset GH deficiency compared with multiple pituitary hormone deficiency (46). These data demonstrate the need to tailor advice for patients based on the aetiology of their GH deficiency.

## Conclusions

As this review has demonstrated, precision medicine can be applied to numerous aspects of the management of endocrine disorders, including biochemical diagnosis, genetic analysis and transitional care. The aim is to personalise care for individual patients by enhancing the quality of diagnosis and treatment. We have demonstrated the contribution of precision medicine to biochemical accuracy in GH assessment, and the ability of precise genetic analysis to confirm pathogenesis and characterise a disorder of puberty and impact on management and therapy. In the transitional phase, accuracy of MRI, together with biochemical precision, can be informative for prognosis and alter therapeutic strategy.

Although in the 20th century, high-quality clinical care attempted to deliver the principles now advocated by precision medicine, real-world evidence suggests that this was not effective. The treatment of all GH-deficient children with the same dose of rhGH was widely practised and the results were not consistently clinically beneficial, with one-third of patients showing poor long-term responses (47). Lack of individualisation of rhGH therapy resulted in up to 40% of patients having subnormal growth responses across a range of licensed rhGH indications (48). Precision medicine provides the framework for a discipline to encourage an updated approach to care.

Over time, the concept of precision medicine has been expanded, not only addressing bio- and genetic markers, but now covering more aspects (3). This evolution reflects the need to achieve personalisation of healthcare that addresses clinical and behavioural elements, in addition to biomedical aspects. This approach is especially important in endocrinology where the environment (e.g. nutrition and physical activity) can have an impact on the disease course for each patient. In order to achieve this, the development of medical informatics and digital health has been seen as a key component in allowing a more holistic view of the patient and, thus, in achieving precision medicine (4).

This review has focused on the role of precision medicine in paediatric and adolescent endocrinology and the role of new digital technologies. For optimal results, precision medicine and associated digital developments/tools must be used in conjunction with a human component. Healthcare professionals, such as nurses or doctors, must receive training in the use of such applications and then incorporate them into their everyday practice.

## Declaration of interest

M B has received research support, travel and accommodation, lecture fees and/or consultancy honoraria from Chiasma, Daisorin, Genexine, GeneScience, IDS, Ionis, Ipsen, Merck, Novartis, OPKO, Pfizer, Roche, Sandoz and StrongBridge. H B has received honoraria for lectures from Novo Nordisk. A C L has no competing interests to declare. M O S has consultancy agreements with Ipsen, Sandoz, Merck KGaA Darmstadt, OPKO and Genexine-Handok; and has received honoraria for lectures from GeneScience, Pfizer and Novo Nordisk.

## Funding

This review is based on a webinar titled ‘Precision Medicine in Endocrine Disorders’ that was presented by the authors on 21 October 2021, which can be viewed at https://paediatric-endocrinology.medwirenews.com/expert-content/webinar/precision-medicine/. The webinar was made possible by an independent educational grant from Merck Healthcare KGaA, Darmstadt, Germany.

## Author contribution statement

M O S was the chair and moderator of the webinar. M B, H G, and A C L were presenters at the webinar*. *All authors read and commented on the drafts of the manuscript, and approved the final version for publication.
